# Identification of Intronless Genes and the Development of KASP Markers for Salt Responses in *Vicia faba* L.

**DOI:** 10.3390/genes17040381

**Published:** 2026-03-27

**Authors:** Jiali Huang, Jinyang Liu, Shuoqian Zhao, Xiaocen Liu, Shengqi Chen, Kailu Zhang, Yun Lin, Qiang Yan, Jingbin Chen, Ranran Wu, Xin Chen, Xingxing Yuan, Yanjie Xie

**Affiliations:** 1 College of Life Sciences, Nanjing Agricultural University, Nanjing 210095, China; 2023816129@stu.njau.edu.cn (J.H.); 18305170275@163.com (X.L.); 2 Institute of Industrial Crops, Jiangsu Academy of Agricultural Sciences, Nanjing 210014, China; 20200024@jaas.ac.cn (J.L.); 20180006@jaas.ac.cn (Y.L.); yanqiang@jaas.ac.cn (Q.Y.); chenjingbin@jaas.ac.cn (J.C.); rrwu@jaas.ac.cn (R.W.); cx@jaas.ac.cn (X.C.); 3 College of Plant Protection, Nanjing Agricultural University, Nanjing 210095, China; 12123518@stu.njau.edu.cn; 4 College of Agriculture, Nanjing Agricultural University, Nanjing 210095, China; 2023801189@stu.njau.edu.cn; 5 State Key Laboratory for Development and Utilization of Forest Food Resources, Co-Innovation Center for Sustainable Forestry in Southern China, State Key Laboratory of Tree Genetics and Breeding, Key Laboratory of State Forestry and Grassland Administration on Subtropical Forest Biodiversity Conservation, College of Life Sciences, Nanjing Forestry University, Nanjing 210037, China; klzhang@njfu.edu.cn

**Keywords:** faba bean, salt stress, intronless gene, transcriptome analysis, KASP

## Abstract

**Background/Objectives:** Salinity stress limits agricultural production and threatens global food security. Faba bean (*Vicia faba* L.) is an important legume crop, and identifying salt-stress-responsive genes may support an improvement in salt response. This study aimed to identify intronless genes in faba bean, screen candidate genes associated with salt-stress responses, and develop a KASP marker for salt-response evaluation. **Methods:** Intronless genes were identified from the faba bean reference genome. Transcriptome analysis was conducted in roots and leaves of two cultivars, Sucan 4 and Yundou 1183, under 150 mM NaCl treatment and control conditions. Candidate genes were examined by expression analysis, functional annotation, PPI prediction, and a luciferase complementation assay. A KASP marker was developed from an SNP within the *VfERF1A* locus and tested in 97 accessions. **Results:** A total of 7581 intronless genes were identified, accounting for 20.69% of annotated genes. Fifteen intronless genes were significantly differentially expressed in both roots and leaves of the two cultivars under salt treatment. Functional annotation suggested that *VfERF1A* and *VfHSP17.8* may be involved in salt-stress responses. PPI prediction and the LUC assay provided preliminary support for a possible association of VfERF1A with VfEIN2. The *VfERF1A*-based KASP marker showed clear genotype clustering, and the two homozygous classes differed significantly in QYmax, relative shoot fresh weight, and relative plant height under salt treatment (*p* < 0.05). The preliminary predictive accuracy for QYmax was 86.36%. **Conclusions:** These results provide a genome-wide resource of intronless genes in faba bean, identify candidate genes associated with salt-stress responses, and describe a preliminary KASP marker associated with salt-response traits. Further validation in independent populations, under diverse environmental conditions, and with additional functional evidence is still required.

## 1. Introduction

Owing to global climate change, improper irrigation, and declining water quality in many agricultural regions, soil salinization has become a major environmental factor limiting agricultural production. Although salinity is generally detrimental to plant growth and development, plant responses may vary with stress intensity, duration, species or genotype, and the traits being evaluated. Therefore, salinity effects may be better understood along response gradients rather than as a strictly binary harmful condition [1]. Plants growing in saline soils need to regulate the uptake and accumulation of Na^+^ and Cl^−^ to maintain ion homeostasis and osmotic balance [2]. This adjustment competes with plant growth for limited energy and resources, leading to significant yield losses [3]. From an agricultural perspective, salt-response phenotyping may therefore be viewed not only as damage assessment, but also as the evaluation of plant performance under defined saline conditions.

Legumes are important food and feed sources which can improve soil fertility and crop productivity through biological nitrogen fixation [4]. Faba bean (*Vicia faba* L.) is an important pulse crop and is significant to human nutrition due to its high level of proteins, fibers, vitamins, and minerals [5]. As a crop used for both food and feed production, faba bean contributes to global food supply, feed resources, and green manure systems [6]. Previous studies have shown that faba bean is highly sensitive to salt stress, and its yield decreases more severely under salinity than that of wheat or maize [7]. This sensitivity is agronomically relevant because salinity in agricultural environments often affects crop growth and productivity across a range of stress intensities. Salt stress inhibits symbiotic nitrogen fixation, weakens osmotic regulation, and prolongs the flowering period, resulting in substantial yield loss. Hydroponic, pot, and field studies have consistently shown significant variation in the responses of faba bean cultivars to salt stress [8,9]. This variation provides a basis for identifying candidate genes associated with salt-stress responses and for evaluating genotypic performance under saline conditions.

In recent years, transcriptomic analyses and genome-wide association studies (GWAS) have identified many genes responsive to salt stress in legumes. In soybean, *GmMYB46* is strongly induced by salt stress, and its overexpression enhances the expression level of salt-responsive genes such as *P5CS1*, *SOD*, *POD*, and *NECD3*, thereby improving salt tolerance [10]. A GWAS of 305 soybean accessions identified a major salt-tolerance locus on chromosome 3 that contains the gene *GmSALT3*, as well as another locus on chromosome 8 associated with Na^+^ and Cl^−^ levels and the chlorophyll ratio [11]. In mung bean, two loci associated with seed germination under salinity were detected on chromosomes 7 and 9 by GWAS [12]. Liu et al. [13] identified a key salt-responsive gene, *VrFRO8*, using GWAS and RNA-seq. This gene improves salt tolerance in mung bean by regulating iron homeostasis and SOD activity. However, due to its very large genome of about 13 Gb, the high proportion of repetitive sequences, limited transformation efficiency, and the complex genetic background of this species, studies on salt tolerance mechanisms in faba bean are still limited compared with other legumes. In addition, as an allogamous species with a complex genetic background, faba bean presents further challenges for functional genomics research. Therefore, identifying informative candidate genes and developing practical molecular markers may provide an efficient strategy for evaluating salt-stress responses and supporting future breeding efforts in faba bean.

In eukaryotes, genes are classified as intron-containing or intronless according to the presence or absence of introns [14]. Intronless genes have a single exon and do not require splicing during transcription, which reduces energy consumption. Intronless genes account for approximately 2.7–97.7% of all genes in eukaryotic genomes [15]. In animals, many intronless genes have been identified, and some of them are highly conserved and function in essential biological processes, while others are related to diseases [16]. In plants, some intronless genes that arise from tandem duplication play important roles in stress responses [17]. Intronless genes are enriched in stress-related functions such as glutathione reductase activity and auxin response in *Poaceae* crops, indicating that they contribute to adaptation to abiotic stresses [18]. Previous studies in plants have suggested that intronless genes may contribute to stress responses, including drought and salinity, and are often enriched in essential metabolic processes [14,19]. However, direct evidence for a more rapid stress responsiveness of intronless genes in legumes remains limited. Therefore, focusing on intronless genes provides a more targeted strategy for identifying salt-stress-responsive candidates in faba bean. Based on these observations, we hypothesized that a subset of intronless genes in *V. faba* may participate in salt-stress responses and could serve as useful candidates for transcriptome-based screening and marker development. However, the repertoire of intronless genes in *V. faba* has not been systematically characterized, and their potential roles in salt-stress responses remain unclear.

Molecular marker technology is an important tool in modern genetics and plays essential roles in crop breeding. Kompetitive Allele-Specific PCR (KASP) is an SNP-based genotyping method characterized by high throughput, high accuracy, low cost, and good flexibility, which makes it suitable for large-scale genotyping in laboratories. KASP markers are widely used in germplasm identification, marker-assisted selection, gene mapping, and seed purity analysis. In soybean, SNPs associated with salt tolerance during germination were converted into KASP markers. These markers effectively distinguished tolerant and sensitive accessions and supported germplasm screening under salt stress [20]. In common wheat, KASP markers targeting salt-affected yield loci enabled accurate allele identification in breeding populations and aided the selection of favorable haplotypes for improved performance in saline environments [21]. In wheat leaves, KASP markers developed from salt-responsive genes identified by transcriptomic and network analyses were validated across diverse germplasm, confirming their association with salt-injury phenotypes and their usefulness in salt-tolerance breeding [22].

Based on the above considerations, in this study, we aimed to systematically characterize intronless genes in faba bean, identify candidate intronless genes associated with salt-stress responses through transcriptome analysis, and develop and evaluate a KASP marker based on a candidate locus. The expression patterns and functional annotations of these genes were analyzed under salt stress. Based on SNPs in candidate genes, a KASP marker was developed and tested in 97 faba bean accessions to evaluate genotype-associated variation in salt-response traits under the tested conditions. This study enhances the understanding of salt-stress responses in faba bean and provides candidate molecular resources that may facilitate the future breeding of salt-tolerant faba bean varieties, contributing to the sustainable development of faba bean production.

## 2. Materials and Methods

### 2.1. Identification of Intronless Genes in Faba Bean

The reference genome sequence and annotation files of *Vicia faba* used in this study were based on the published Hedin/2 chromosome-scale assembly reported by Jayakodi et al. [23] and were obtained from the Fabagenome database (https://projects.au.dk/fabagenome/genomics-data, accessed on 30 August 2025). Single-exon genes were identified as intronless from the genome annotation file. To reduce the inclusion of likely annotation artifacts, only annotated protein-coding genes were retained for further analysis. The number, gene length, and chromosomal location of intronless genes were summarized using Microsoft Excel (2019), and their chromosomal distribution was visualized using Tbtools v2.441.

### 2.2. Plant Materials

Two *Vicia faba* cultivars with different salt sensitivities, Yundou 1183 (salt-sensitive) and Sucan 4 (salt-tolerant), were provided by the Institute of Industrial Crops, Jiangsu Academy of Agricultural Sciences, Nanjing, China. Seeds were surface-sterilized with 10% (*v*/*v*) NaClO, rinsed, soaked for 12 h, and germinated on moist filter paper. Seedlings were then transplanted into vermiculite-filled pots (13 cm in upper diameter and 12 cm in height) and grown at 23 °C/18 °C (day/night) under a 16 h/8 h photoperiod [24]. During cultivation, seedlings were watered with distilled water. The seedlings were irrigated with 150 mM NaCl solution for 10 days starting at 12 days after germination, when the first pair of leaves were fully expanded, with treatments applied every two days [25]. Salt stress was imposed abruptly by directly applying 150 mM NaCl solution until leachate appeared from the bottom of the pot. No EC monitoring was performed during the treatment period. A single salinity level (150 mM NaCl) was used to provide a clearly discriminating stress condition for transcriptome comparison between contrasting cultivars, rather than to define the full response pattern across salinity gradients. Each treatment included three biological replicates, and each biological replicate consisted of pooled tissues from five plants. Root and leaf samples were collected at the same sampling time point after 10 days of salt treatment for transcriptome sequencing. Whole-root tissues and young leaves were sampled.

### 2.3. RNA-Seq and Functional Annotation

Total RNA quality was examined using a kaiaoK5500 spectrophotometer (Kaiao, Beijing, China) and Agilent 2100 Bioanalyzer system (Agilent Technologies, Santa Clara, CA, USA). High-quality RNA was used for mRNA enrichment, fragmentation, and cDNA synthesis. RNA-seq libraries were constructed following the standard protocol of Wuhan Benagen Technology Co., Ltd., Wuhan, China. and were sequenced on the MGI T7 platform (MGI Tech, Shenzhen, China) to generate 150 bp paired-end reads. Raw reads were filtered using fastp (v0.21.0) to remove adapters and low-quality sequences, and read quality was assessed using FastQC (v0.11.9; default parameters). Clean reads were aligned to the *Vicia faba* reference genome using STAR (v2.7.9a; default parameters). Gene expression levels were quantified with RSEM (v1.3.1) and expressed as FPKM values. Differentially expressed genes were identified using DESeq2, and *p* values were adjusted using the Benjamini–Hochberg method to control the false discovery rate (FDR). Genes with FDR < 0.05 and |log_2_FoldChange| > 1.0 were defined as differentially expressed genes. The sequencing quality and mapping statistics for all RNA-seq libraries, including raw and clean read counts, Q30 values, GC content, and mapping rates, are summarized in Table S1. Gene Ontology (GO) and Kyoto Encyclopedia of Genes and Genomes (KEGG) enrichment analyses were performed using the clusterProfiler package. Differential expression analysis was performed on all detected transcripts, including annotated genes and novel transcripts identified in the RNA-seq data.

### 2.4. qRT-PCR Analysis

To validate the RNA-seq results, quantitative real-time PCR (qRT-PCR) was performed using the same RNA samples. First-strand cDNA was synthesized using the HiScript III 1st Strand cDNA Synthesis Kit (R312-02, Vazyme., Nanjing, China), and qRT-PCR was performed using ChamQ SYBR qPCR Master Mix (Vazyme Biotech, Nanjing, China) on a LightCycler 480 II Real-Time PCR System (Roche, Basel, Switzerland). The cycling program was: 95 °C for 3 min, followed by 30 cycles of 95 °C for 30 s, 55 °C for 30 s, and 72 °C for 30 s, with a final extension at 72 °C for 5 min. The *VfCYP2* gene was used as an internal reference gene [26]. Primer sequences are provided in Table S2. Three biological replicates were analyzed for each treatment, and each qRT-PCR reaction was performed with three technical replicates. The relative expression levels were quantified by the 2^−ΔΔCT^ method.

### 2.5. Prediction of Protein–Protein Interaction

To predict the potential functions of the candidate intronless genes in salt-stress response networks, protein–protein interaction (PPI) analysis was conducted. The amino acid sequences of *VfHSP17.8* and *VfERF1A* were aligned with the *Arabidopsis thaliana* proteome (Araport11, accessed on 14 May 2025) using BLASTP (Tbtools v2.441) to identify orthologs. A preliminary set of salt-related *Arabidopsis* proteins was assembled for exploratory network construction. Salt-related *Arabidopsis* proteins were collected from the TAIR database (https://www.arabidopsis.org/) using “salt” and “salt stress” as keywords and supplemented with known components of salt-stress regulatory pathways, including the SOS pathway, ABI transcription factors, and CBL/CIPK and CDPK kinase families. The *Arabidopsis* orthologs of *VfHSP17.8* and *VfERF1A* and these salt-responsive proteins were submitted to the STRING v11.5 database (https://string-db.org/, accessed on 20 May 2025) for PPI prediction. A relatively low confidence score threshold (>0.15) was used for exploratory network construction in order to capture potentially relevant associations between the candidate genes and known salt-stress regulators. The resulting interaction networks were visualized in Cytoscape (v3.10.3). The retained protein IDs used for this analysis and their basis for inclusion are provided in Table S3.

### 2.6. Yeast Two-Hybrid Assay

The coding sequence (CDS) of *VfERF1A* was amplified from faba bean cDNA and cloned into the pGADT7 vector to generate the prey construct fused with the GAL4 activation domain (AD). The full-length CDSs of the putative EIN2- and S6K2-like homologs identified in the faba bean genome were amplified and inserted into the pGBKT7 vector to generate bait constructs fused with the GAL4 DNA-binding domain (BD). All recombinant plasmids were verified by sequencing and co-transformed into the yeast strain AH109 using the lithium acetate method. Transformants were first selected on SD medium lacking tryptophan and leucine (SD/-Trp/-Leu) to confirm successful co-transformation. Autoactivation tests were performed using bait + empty prey and prey + empty bait combinations before interaction assessment. The positive and negative controls supplied with the Matchmaker system (pGBKT7-53/pGADT7-T and pGBKT7-Lam/pGADT7-T) were included in the assay. Protein–protein interactions were assessed on SD medium lacking tryptophan, leucine, histidine, and adenine (SD/-Trp/-Leu/-His/-Ade) after incubation at 30 °C for 3–5 days. Because weak background activation was observed in some candidate combinations, the resulting Y2H data were treated only as preliminary supplementary evidence and were interpreted cautiously.

### 2.7. Luciferase Complementation Imaging Assay

To further examine the predicted interaction between VfERF1A and VfEIN2, a luciferase complementation imaging (LCI) assay was performed. The coding sequence of *VfERF1A* was cloned into the pCAMBIA1300-cLUC vector to generate the cLUC-VfERF1A construct, while the coding sequence of *VfEIN2* was cloned into the pCAMBIA1300-nLUC vector to generate the nLUC-VfEIN2 construct. All recombinant plasmids were confirmed by sequencing. The recombinant plasmids were transformed into *Agrobacterium tumefaciens* strain GV3101 and transiently co-expressed in Nicotiana benthamiana leaves. Two days after infiltration, leaves were treated with 150 µM luciferin (Coolaber, Beijing, China), and luminescence signals were captured using a Tanon 3500 imaging system (Tanon, Shanghai, China) under dark conditions with an exposure time of 10 min. Luminescence signals were evaluated qualitatively by visual comparison with the negative controls. The assay was performed with three biological replicates, and no formal luminescence quantification or statistical analysis was conducted. AVH69-nLUC + LRR-cLUC was used as a positive control, whereas VfERF1A-cLUC + nLUC and VfEIN2-nLUC + cLUC were used as negative controls. Primer sequences are provided in Table S4.

### 2.8. Measurement of Chlorophyll Fluorescence Parameters

To evaluate salt tolerance among faba bean accessions, the maximum photochemical efficiency of photosystem II (QYmax = Fv/Fm) was measured 7 days after salt treatment using a FluorCam 7 portable chlorophyll fluorescence imaging system (Photon Systems Instruments, Drásov, Czech Republic). Before measurement, plants were dark-adapted for 1 h to ensure the full opening of the reaction centers. For each accession, three biological replicates were used, with one plant serving as one biological replicate, and the mean value was used for analysis. In each replicate, the first fully expanded pair of leaves from the bottom of the plant was selected for QYmax measurement. Each pot containing one plant was considered one biological replicate. Before measurement, the 97 accessions were coded numerically, and measurements were performed according to these codes rather than the cultivar identities. Higher QYmax values indicate lower PSII damage and better physiological performance under the tested salt treatment. QYmax was therefore used as a physiological index of salt response for association analysis with the KASP genotypes.

### 2.9. Development of KASP Markers

KASP genotyping was conducted using HiGeno 2× Probe Mix A (Beijing Jiacheng Biotechnology Co., Ltd., Beijing, China) in a 5 μL reaction system on a QuantStudio™ 7 Flex System (Thermo Fisher Scientific, Waltham, MA, USA). The allele-specific primers were designed with universal fluorescent tags (FAM and VIC) at their 5′ ends. Primers were diluted to 10 μM and mixed at a ratio of 1:1:3 (allele-specific primer 1: allele-specific primer 2: common primer). Each reaction contained 2.5 μL 2× KASP Master Mix, 1.25 μL primer mix, and 1.25 μL genomic DNA. Reactions were dispensed into 384-well plates, sealed, vortexed, and centrifuged before PCR. The cycling program consisted of 95 °C for 10 min; 10 touchdown cycles of 95 °C for 20 s and 61–55 °C for 60 s; followed by 27 cycles of 95 °C for 20 s and 55 °C for 60 s; and a final fluorescence reading at 25 °C for 30 s. Primer sequences used for KASP genotyping are listed in Table S5. A total of 97 faba bean accessions were genotyped, and three genotype classes (AA, AC, and CC) were identified. Because the heterozygous group (AC) was relatively small and showed greater phenotypic variation, the main statistical comparisons of salt-response traits were conducted between the two homozygous groups using an independent-samples *t*-test at *p* < 0.05. To reduce the risk of inflated type I error across multiple trait comparisons, the resulting *p* values were further adjusted using the Benjamini–Hochberg false discovery rate (FDR) procedure.

The predictive accuracy of the KASP marker was evaluated based on QYmax values under salt treatment. The 97 accessions were ranked according to QYmax, and the highest 30% (n = 29) and lowest 30% (n = 29) were defined as the relatively salt-tolerant and relatively salt-sensitive groups under the tested conditions, respectively, while the remaining accessions were considered intermediate. The AA genotype was regarded as the salt-tolerance-associated genotype, and the CC genotype as the salt-sensitive-associated genotype. Marker accuracy was calculated as the proportion of homozygous accessions in the two extreme phenotype groups whose genotype-based classification was consistent with their phenotypic grouping. Predictive accuracy (%) = (number of correctly classified homozygous accessions/total number of homozygous accessions in the two extreme phenotype groups) × 100. This value was used as a preliminary within-panel estimate of marker performance under the tested conditions.

## 3. Results

### 3.1. Genomic Distribution and Structural Features of Intronless Genes in Faba Bean

A total of 7581 intronless genes were identified in the *Vicia faba* genome, accounting for 20.69% of all annotated genes and distributed across seven chromosomes (Table S6). On average, each chromosome contained 1083 intronless genes, but the numbers varied among chromosomes. Chromosome 5 had the fewest intronless genes (839), whereas chromosome 1 L had the most (1471). The mean length of all annotated genes in the genome was approximately 2919 bp, while intronless genes averaged 841 bp, about one-third of the genome-wide average. Thus, intronless genes in faba bean are shorter and more compact.

**Figure 1:**
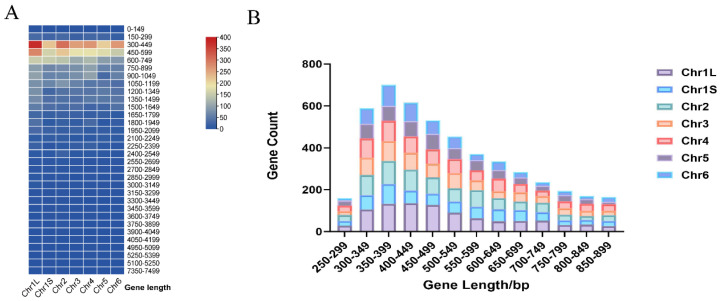
Length distribution of intronless genes in *Vicia faba*. (**A**) Overall length distribution of all intronless genes. (**B**) Distribution of intronless genes ranging from 250 to 899 bp.

Gene length distribution analysis showed that most intronless genes ranged from 300 to 900 bp (Figure 1A). Genes shorter than 1350 bp accounted for 83% of all intronless genes, and 98% were shorter than 2550 bp, indicating that most intronless genes are relatively short. Only 1% exceeded 2850 bp, representing a very small proportion of long genes. A more detailed analysis of genes between 250 and 899 bp, using 50 bp intervals, revealed that those in the 350–400 bp range were the most abundant (Figure 1B). These results show that intronless genes in *V. faba* are compact, short, and concentrated within a narrow length range.

### 3.2. Differential Expression and Functional Enrichment of Total DEGs Under Salt Treatment

**Figure 2:**
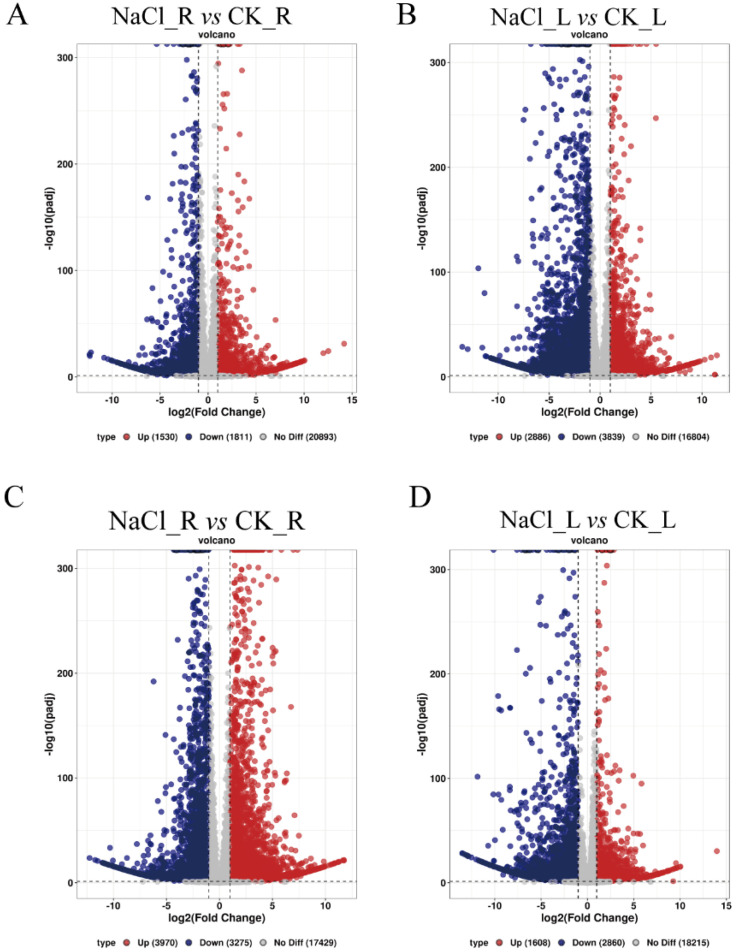
Volcano plots of differentially expressed genes (DEGs) between salt-treated and control samples. (**A**) Roots of Yundou 1183. (**B**) Leaves of Yundou 1183. (**C**) Roots of Sucan 4. (**D**) Leaves of Sucan 4. Red dots indicate upregulated genes (log_2_FC > 1, Padj < 0.05), blue dots indicate downregulated genes (log_2_FC < −1, Padj < 0.05), and gray dots represent genes with no significant expression changes. Dotted lines indicate log_2_FC = 0 and −log10(padj) = 1.3 (corresponding to Padj = 0.05).

Principal component analysis (PCA) revealed clear separation between salt-treated and control samples in both cultivars (Figure S1). Using Padj < 0.05 and |log_2_FC| > 1 as thresholds, differentially expressed genes responsive to salt stress were identified in both cultivars. FPKM values for all genes across all samples are provided in Supplementary Table S7. A summary of the DEGs identified in the four pairwise comparisons is provided in Table S8. Differentially expressed genes (DEGs) varied substantially between tissues and cultivars, with their numbers summarized in Figure S2. In Yundou 1183, 1530 genes were upregulated and 1811 were downregulated in roots, while 2886 genes were upregulated and 3839 were downregulated in leaves (Figure 2A,B). In Sucan 4, 3970 genes were upregulated and 3275 were downregulated in roots, and 1608 genes were upregulated and 2860 were downregulated in leaves (Figure 2C,D). These DEG counts suggest tissue- and cultivar-dependent differences in transcriptomic responsiveness under salt stress, with leaves of Yundou 1183 and roots of Sucan 4 showing larger numbers of differentially expressed transcripts. Because the counts included both annotated genes and novel transcripts, these differences were interpreted cautiously, and biological interpretation was based primarily on expression patterns and functional enrichment rather than DEG number alone. These patterns were further supported by the hierarchical clustering of DEGs, which revealed a clear grouping of samples according to tissue type and treatment (Figure S3).

**Figure 3:**
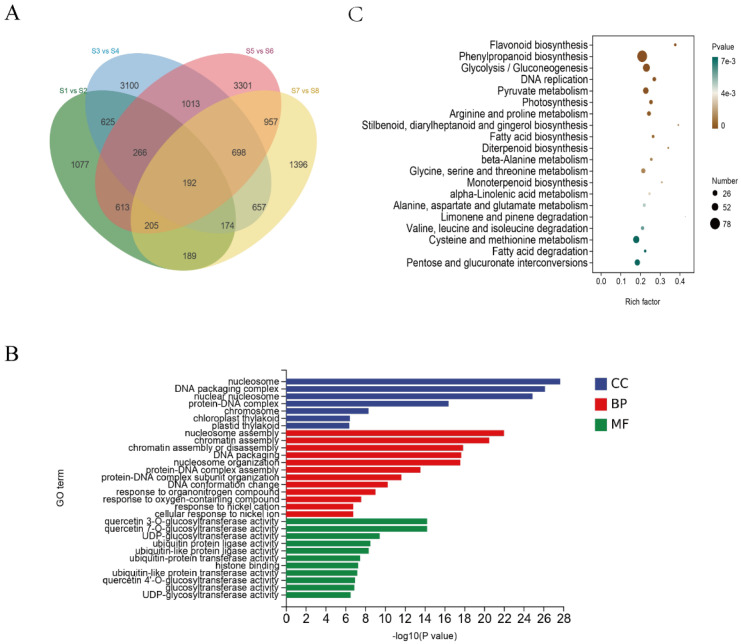
Analysis of differentially expressed genes (DEGs) under salt stress. (**A**) Venn diagram showing the overlap of DEGs in roots and leaves of Yundou 1183 and Sucan 4. (**B**) Gene Ontology (GO) enrichment analysis of DEGs. (**C**) KEGG pathway enrichment analysis of DEGs. S1–S8 represent the control and 150 mM NaCl-treated roots and leaves of Yundou 1183 and Sucan 4, respectively.

A total of 192 DEGs were shared across both cultivars and both tissues under salt treatment (Figure 3A). GO enrichment analysis showed that the DEGs were mainly involved in chromatin assembly, nucleosome organization, and protein–DNA complex assembly in the Biological Process category. In the Molecular Function category, they were enriched in UDP-glucosyltransferase and ubiquitin-protein ligase activities, and in the Cellular Component category, they were associated with the nucleosome, DNA packaging complex, and chromosomal structures (Figure 3B). KEGG analysis showed significant enrichment in flavonoid and phenylpropanoid biosynthesis, as well as pathways related to arginine and proline metabolism, alanine–aspartate–glutamate metabolism, glycolysis/gluconeogenesis, pyruvate metabolism, fatty acid degradation, and α-linolenic acid metabolism (Figure 3C).

### 3.3. Differential Expression and Functional Enrichment of Intronless Genes

**Figure 4:**
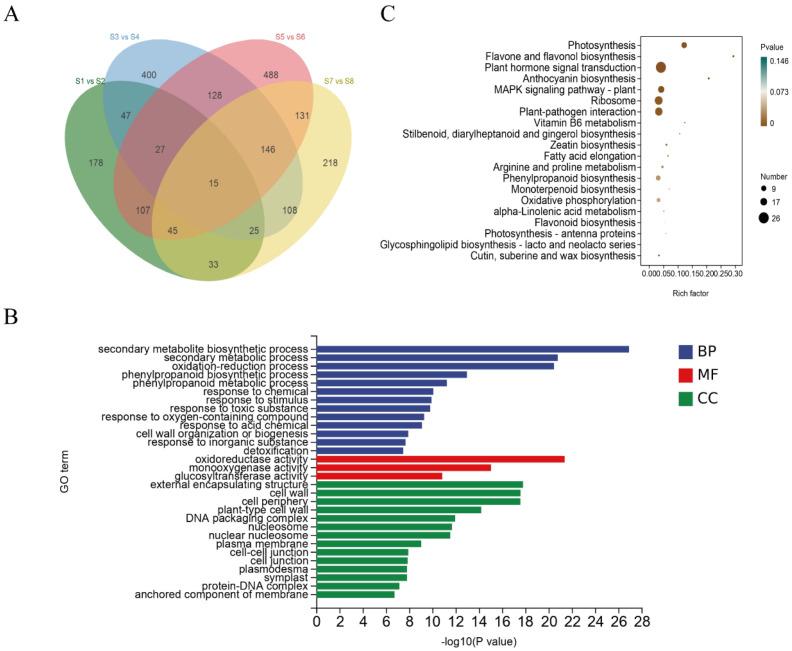
Analysis of intronless differentially expressed genes (DEGs) under salt stress. (**A**) Venn diagram of intronless DEGs in roots and leaves of *Yundou 1183* and *Sucan 4*. (**B**) GO enrichment analysis of intronless DEGs. (**C**) KEGG enrichment analysis of intronless DEGs.

A total of 15 intronless DEGs were commonly responsive across the roots and leaves of both Yundou 1183 and Sucan 4 under salt treatment (Figure 4A; Table S9). All of these intronless DEGs met the thresholds of |log_2_FC| > 1 and Padj < 0.05.

GO enrichment analysis showed that the intronless DEGs were mainly involved in secondary metabolite biosynthetic processes, phenylpropanoid metabolism, and oxidation–reduction processes in the Biological Process category. In the Molecular Function category, they were enriched in glucosyltransferase and oxidoreductase activities, and in the Cellular Component category, they were associated with the cell wall, DNA packaging complex, and nucleosome (Figure 4B). KEGG enrichment analysis revealed that intronless DEGs were significantly enriched in flavone and flavonol biosynthesis, anthocyanin biosynthesis, and phenylpropanoid biosynthesis. Additional enriched pathways included plant hormone signal transduction, MAPK signaling, arginine and proline metabolism, photosynthesis, and oxidative phosphorylation (Figure 4C).

A comparative analysis of all DEGs and intronless DEGs showed that both groups were associated with major salt-responsive pathways, including reactive oxygen species detoxification, ion homeostasis, and secondary metabolism. Intronless DEGs exhibited a higher proportion of genes related to cell wall remodeling, ion transport, and chromatin-associated processes, while all DEGs showed broader representation in plant hormone signaling and transcriptional regulatory networks. Under salt stress, *Vfaba.Tiffany.R1.1g199440.1* showed 64% amino acid sequence similarity to the Arabidopsis salt-responsive gene *AtSZF1*. Previous studies have reported that *AtSZF1* is rapidly induced by salt treatment and contributes to salt tolerance regulation [27]. The intron-containing gene *Vfaba.Tiffany.R1.2g194320.2* exhibited 71% sequence similarity to *AtP5CS1*, which is known to promote proline accumulation under salt stress in Arabidopsis [28]. These results suggest that both types of genes may play important roles in the salt-stress response of faba bean.

### 3.4. Validation of Candidate Intronless Genes by qRT-PCR

**Figure 5:**
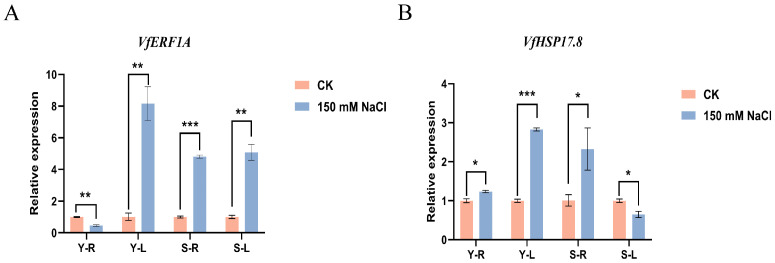
qRT-PCR validation of two candidate intronless genes under salt stress. (**A**) Relative expression of *VfERF1A* in roots and leaves of Yundou 1183 and Sucan 4. (**B**) Relative expression of *VfHSP17.8* in roots and leaves of Yundou 1183 and Sucan 4. Values are shown as mean ± SD of three biological replicates, each with three technical replicates. Error bars represent SD. Asterisks indicate significant differences between CK and 150 mM NaCl treatment within the same tissue/cultivar, as determined by Student’s *t*-test (* *p* < 0.05, ** *p* < 0.01, *** *p* < 0.001).

Functional annotation of the 15 intronless DEGs identified under salt stress highlighted two genes, *Vfaba.Tiffany.R1.4g106080.1* and *Vfaba.Tiffany.R1.6g083720.1*, which showed high sequence similarity to *Arabidopsis thaliana AT4G17500 (ERF1A)* and *AT1G07400 (HSP17.8)*, respectively. These genes were designated *VfERF1A* and *VfHSP17.8*. To evaluate the RNA-seq expression patterns, qRT-PCR was performed using the same RNA samples as those used for transcriptome sequencing (Figure 5).

For *VfERF1A*, qRT-PCR results closely mirrored the RNA-seq trends across tissues and cultivars, revealing clear tissue- and cultivar-dependent regulation under salt treatment. Specifically, *VfERF1A* was upregulated in both roots and leaves of Sucan 4, whereas in Yundou 1183 it was downregulated in roots but upregulated in leaves. The concordant results between RNA-seq and qRT-PCR support the robustness of the *VfERF1A* expression pattern under salt stress.

For *VfHSP17.8*, both RNA-seq and qRT-PCR consistently showed strong induction in roots of both cultivars under salt treatment, indicating a conserved root-responsive pattern. In leaves, *VfHSP17.8* displayed cultivar-dependent regulation. In Sucan 4 leaves, both RNA-seq and qRT-PCR indicated downregulation. However, in Yundou 1183 leaves, RNA-seq indicated downregulation, but qRT-PCR indicated upregulation. This inconsistency was therefore most evident in the Y-L group and may reflect differences in detection sensitivity, transcript abundance, or other methodological differences between RNA-seq and qRT-PCR.

Overall, these results indicate that *VfERF1A* and *VfHSP17.8* show cultivar-specific and tissue-specific transcriptional responses to salt stress. Compared with *VfERF1A*, *VfHSP17.8* showed a less consistent expression pattern in Yundou 1183 leaves between the two assays, and this result should be interpreted with caution.

### 3.5. PPI Prediction of Candidate Salt-Stress-Related Genes

**Figure 6:**
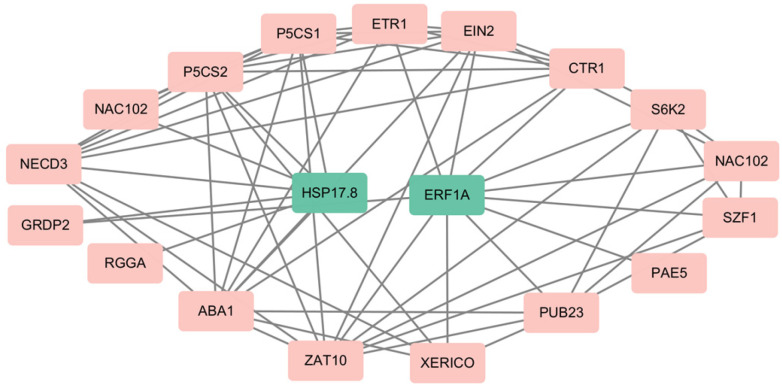
Predicted protein–protein interaction (PPI) networks of candidate salt-stress-related genes. Predicted interaction networks of *VfERF1A* and *VfHSP17.8* based on their Arabidopsis orthologs and known salt-stress-related proteins.

A protein–protein interaction (PPI) analysis was conducted to explore potential functional associations of *VfERF1A* and *VfHSP17.8* under salt stress (Figure 6; Table S10). For *VfHSP17.8*, the predicted network included proteins associated with proline biosynthesis and abscisic acid (ABA) metabolism, such as *P5CS1/2* and *ABA1* [28,29]. In contrast, the predicted network of *VfERF1A* contained multiple signaling regulators and components of the ethylene pathway, including *ETR1*, *CTR1*, and *EIN2* [30], suggesting a possible signaling context for *VfERF1A* in salt-stress-related responses. Preliminary yeast two-hybrid (Y2H) observations for selected candidate interactions are presented in Supplementary Figure S4. Because weak background activation was observed in the negative controls, these Y2H results are treated only as supplementary evidence and should be interpreted with caution.

### 3.6. LUC Assay Supporting the Interaction Between VfERF1A and VfEIN2

**Figure 7:**
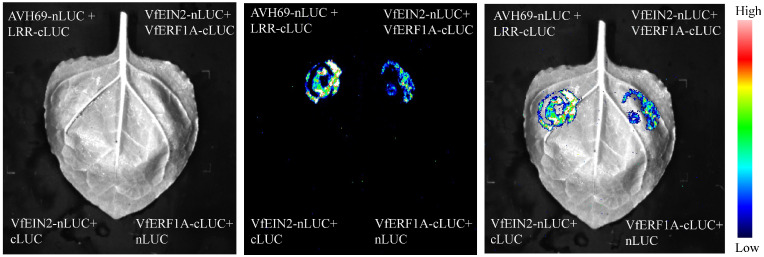
LUC assay supporting the interaction between VfERF1A and VfEIN2. Luciferase complementation imaging (LCI) assay in Nicotiana benthamiana leaves showing the interaction between VfERF1A-cLUC and VfEIN2-nLUC. The co-expression of VfERF1A-cLUC and VfEIN2-nLUC produced a visible luminescence signal, whereas the negative controls VfERF1A-cLUC + nLUC + nLUC and VfEIN2-nLUC + cLUC did not show a detectable signal. AVH69-nLUC + LRR-cLUC was used as a positive control.

To further examine the predicted association between VfERF1A and VfEIN2, a luciferase complementation imaging (LCI) assay was performed in *Nicotiana benthamiana* leaves. The co-expression of VfERF1A-cLUC and VfEIN2-nLUC produced a clear luminescence signal, whereas the negative-control combinations VfERF1A-cLUC + nLUC and VfEIN2-nLUC+ cLUC did not show detectable signal (Figure 7). The positive control (AVH69-nLUC + LRR-cLUC) showed the expected strong luminescence signal. These results provided additional experimental support for the interaction between VfERF1A and VfEIN2.

### 3.7. KASP Marker Development for Salt Response

To develop a KASP marker associated with salt-stress response in faba bean, multiple SNP loci were surveyed within the *VfERF1A* region (Table S11). An A/C SNP located at chr4-919,763,421 was selected to design a kompetitive allele-specific PCR (KASP) assay.

**Figure 8:**
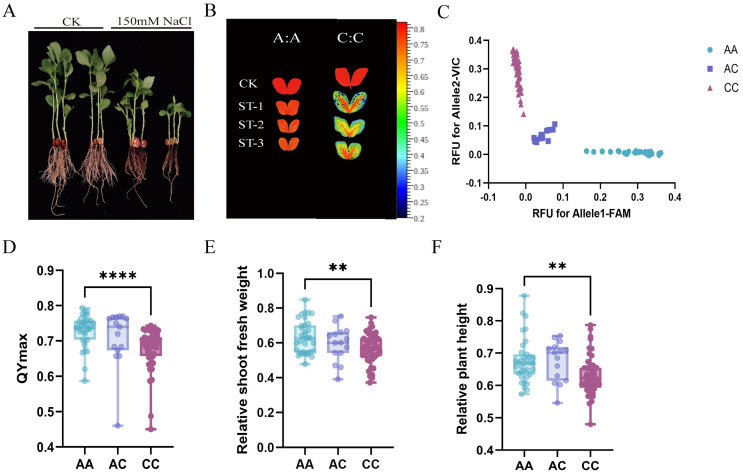
Evaluation of the *VfERF1A*-based KASP marker for salt-stress response in faba bean. (**A**) Representative seedlings of the AA and CC genotypes under control (CK) and 150 mM NaCl treatment. (**B**) Chlorophyll fluorescence imaging (Fv/Fm) of representative seedlings under CK and salt stress. (**C**) KASP allele discrimination plot showing the distribution of the 97 accessions into three genotype classes: AA (n = 35), AC (n = 15), and CC (n = 47). The frequency of the A allele was 0.438, and that of the C allele was 0.562, while the observed heterozygosity was 0.155 in the 97-accession panel. RFU, relative fluorescence units. (**D**–**F**) Comparisons of salt-response traits under salt stress, including (**D**) QYmax (Fv/Fm), (**E**) relative shoot fresh weight (salt/CK), and (**F**) relative plant height (salt/CK). Values are shown as mean ± SD. The AC genotype group was retained in the genotyping results, but the main statistical analyses of salt-response traits were focused on the AA and CC groups. Statistical significance was determined using an independent-samples *t*-test between the AA and CC groups (** *p* < 0.01, **** *p* < 0.0001).

KASP genotyping of the 97-accession panel separated the accessions into three genotype classes (AA, n = 35; AC, n = 15; CC, n = 47) with clear clustering, including a distinct heterozygote group (Figure 8C; Table S12). Seedlings were then subjected to 150 mM NaCl, and salt-response-related traits were assessed. Representative seedlings of the AA and CC genotypes are shown in Figure 8A. Chlorophyll fluorescence imaging indicated higher QYmax (Fv/Fm) in the AA genotype compared with the CC genotype under salt stress (Figure 8B). In addition, we calculated relative shoot fresh weight and relative plant height as the ratio of the value under salt treatment to the corresponding control (salt/CK) (Figure 8D–F).

The AC genotype group was retained in the genotyping results and showed clear clustering, but its phenotypic values were generally intermediate and more variable than those of the two homozygous groups. Therefore, the main comparisons of salt-response traits were focused on the two homozygous genotype classes. Under salt treatment, *t*-tests revealed significant differences between the two homozygous genotype classes (AA vs. CC) for QYmax (Fv/Fm), relative shoot fresh weight, and relative plant height (salt/CK) (*p* < 0.05). The differences between the two homozygous genotype groups remained significant after Benjamini–Hochberg FDR correction for multiple trait comparisons. Based on QYmax ranking under salt treatment, the highest 30% (n = 29) and lowest 30% (n = 29) of accessions were defined as the salt-tolerant and salt-sensitive groups, respectively. Among the homozygous accessions in these two extreme phenotype groups, the preliminary predictive accuracy of the *VfERF1A*-based KASP marker was 86.36%.

These results indicate that the *VfERF1A*-based KASP marker is associated with variation in salt-stress response traits in the tested panel and may be useful for distinguishing contrasting salt-response phenotypes in the tested panel.

## 4. Discussion

Salinity is an important constraint to crop growth and productivity [31]. However, the magnitude and pattern of plant responses to salinity may vary with stress severity, exposure conditions, genotype, and the traits being evaluated. Faba bean (*Vicia faba* L.), an important legume for human nutrition and sustainable farming systems, is particularly sensitive to adverse environmental conditions [32]. In this context, the present study extends the current understanding of salt-stress responses in *V. faba* by integrating the genome-wide identification of intronless genes, transcriptome-based candidate prioritization, and marker evaluation under a defined salt treatment. In particular, we identified 7581 intronless genes in the faba bean genome and found that a subset of these genes showed tissue- and cultivar-dependent responses under the tested conditions. These findings provide a focused framework for examining the potential roles of intronless genes in salt-stress responses under defined experimental conditions and for prioritizing candidate genes for subsequent functional analysis and marker evaluation in faba bean.

In this study, genome-wide analysis identified 7581 intronless genes in the *V. faba* genome, accounting for 20.69% of annotated genes. Their average length was markedly shorter than that of typical intron-containing genes, reflecting a compact structural organization. This observation is consistent with the findings of Jain et al. [33] in rice and *Arabidopsis*, where intronless genes were shown to encode shorter proteins. Such compact gene architecture may contribute to their ability to respond rapidly to environmental stresses. Transcriptome analysis revealed cultivar- and tissue-dependent responses to salt stress in faba bean. Notably, Sucan 4 roots contained the largest number of differentially expressed genes among the tissue–cultivar comparisons. This pattern aligns with the adaptive strategy of salt-tolerant genotypes, in which roots function as the primary site for sensing salinity and initiating early mechanisms related to ion homeostasis [34]. These patterns provide a useful resource for prioritizing candidate genes and for subsequent marker development and evaluation.

GO and KEGG enrichment analyses indicated that salt stress activated pathways related to secondary metabolism in faba bean, particularly phenylpropanoid biosynthesis and downstream branches such as flavonoid and anthocyanin metabolism. Products of these pathways are widely associated with stress mitigation, as they can strengthen cell-wall structure and enhance antioxidant capacity. For example, lignin deposition contributes to cell-wall reinforcement, whereas flavonoids can act as non-enzymatic antioxidants that help limit the accumulation of reactive oxygen species (ROS) under stress [35,36]. Flavonoids derived from this pathway can function as non-enzymatic antioxidants, directly scavenging ROS generated under salt stress and mitigating oxidative injury. This role helps maintain cellular homeostasis and contributes to improved salt tolerance [37]. However, because this study did not directly measure metabolite accumulation, ROS dynamics, ion fluxes, or related physiological parameters, these interpretations remain inferential and require further experimental validation. Taken together, these results may be broadly consistent with major physiological components of salt-stress responses, including ROS detoxification, osmotic adjustment, and ion homeostasis.

*VfHSP17.8* identified in this study belongs to the small heat shock protein (HSP20) family, a group widely recognized for its conserved roles in plant adaptation to abiotic stress. Proteins in this family often function as molecular chaperones that stabilize unfolded proteins and maintain cellular homeostasis under stress conditions. Consistent with this role, Wang et al. [38] showed that *TaHSP17.4* enhances wheat tolerance to drought, salinity, and heat by interacting with TaHOP and modulating ROS scavenging and ABA-related signaling pathways. Cai et al. further demonstrated that the overexpression of tomato *SlHSP17.3* in *Arabidopsis* increases antioxidant enzyme activities and induces key stress-responsive genes, including *AtCOR15*, *AtDREB1B*, and *AtHSFA2*, thereby strengthening salt tolerance [39]. Similarly, Zhang et al. reported that the cytosolic class II sHSP *PfHSP17.2* from *Primula forrestii* confers enhanced resistance to heat, cold, and salt stress when overexpressed in *Arabidopsis* [40]. Together, these studies support the view that HSP20 family members are often associated with abiotic-stress responses. In the present study, the salt-responsive expression of *VfHSP17.8* and its orthology-based annotation suggest that it may participate in stress-related responses in faba bean. However, because no direct physiological or biochemical measurements were performed here, the underlying mechanism remains to be clarified.

In parallel, *VfERF1A* identified in this study belongs to the AP2/ERF transcription factor family, whose members are well known for mediating plant responses to abiotic stress. The AP2/ERF family, particularly the DREB1/CBF and DREB2 subgroups, plays central roles in plant responses to cold, drought, and heat stresses by activating downstream genes via binding to DRE/CRT cis-elements [41]. Although *ERF1A* has not previously been directly linked to salt-stress responses, its differential expression under NaCl treatment in this study suggests that it may be involved in salt-related signaling. Evidence from other species supports this possibility. Nasiopoulou et al. [42] verified that *ERF1A* in peach fruit responds to UV-C stress by regulating salicylic acid signaling, cell-wall metabolism, and flavonoid biosynthesis to maintain cellular homeostasis. In *Arabidopsis*, *ERF1A* functions as a substrate of MPK3/MPK6 and participates in a feedback loop that modulates the expression of key ethylene biosynthesis genes, including *ACS2* and *ACS6*, during biotic stress responses [43]. In our study, the predicted interaction network suggested a possible association of VfERF1A with VfEIN2 and VfS6K2. Importantly, the LUC assay further supported the interaction between VfERF1A and VfEIN2, providing additional experimental support for a possible ethylene-related signaling context. By contrast, because weak background activation was observed in the negative controls of the Y2H assay, those results were treated more conservatively and are presented only as supplementary evidence (Figure S4). Taken together, the available evidence suggests that *VfERF1A* may be involved in salt-related signaling, but these observations do not by themselves establish a direct causal role in salt tolerance, and further functional validation will be required.

From an applied perspective, the *VfERF1A*-based KASP marker produced clear genotype clustering and was associated with variation in multiple salt-response traits in the tested panel under the present experimental conditions, indicating its potential utility for salt-response evaluation under the present conditions. Because the present study was conducted under a single, relatively strong salinity regime (150 mM NaCl), these results should be interpreted as comparative performance under that specific condition rather than as a complete characterization of genotype-specific responses across broader agronomic salinity gradients. The AC group was retained in the genotyping results, but its phenotypic values were generally more variable and less clearly separated than those of the two homozygous groups. Therefore, under the present conditions, the marker was more informative for distinguishing contrasting homozygous genotype classes than for interpreting heterozygous accessions. Based on QYmax, the marker showed a predictive accuracy of 86.36% in the tested panel, indicating that genotype–phenotype inconsistency remained for a subset of accessions. In this sense, the marker should be viewed as supportive rather than definitive for phenotype inference. Moreover, the tested panel was relatively small, which limits the statistical power of the association analysis. The phenotypic evaluation was conducted under a specific experimental condition, and the transferability of the marker across broader genetic backgrounds and environments remains to be tested. From a breeding perspective, the present result is consistent with previous studies showing that KASP markers can serve as useful tools for salt-related trait evaluation and marker-assisted selection in crop improvement [20,21,22]. Additional validation in independent populations and under diverse environmental conditions will be necessary before broader practical application can be considered.

Overall, this study provides a genome-wide catalogue of intronless genes in faba bean, identifies transcriptome-supported salt-stress-responsive candidates, and reports a *VfERF1A*-based KASP marker associated with variation in salt-response traits. However, several limitations should be acknowledged. These findings were derived from transcriptome comparisons between only two cultivars and from marker development based on a single candidate locus under a relatively strong experimental salt treatment. In addition, the phenotypic evaluation was conducted at a single defined time point, and the validation panel was relatively small, which limits the statistical power and broader generalizability of the association analysis. Therefore, the present results should not be generalized to faba bean salt tolerance more broadly without additional evidence from larger and independent populations, additional loci, and diverse environmental conditions. The combined evidence from expression analysis, interaction prediction, and the LUC assay supports the possible involvement of VfERF1A in stress-related signaling, whereas the Y2H result is retained only as cautious supplementary evidence because of weak background activation in the negative controls. Despite these limitations, the present study provides useful resources for future investigations of salt-stress responses in faba bean and may support subsequent marker evaluation in broader breeding materials.

## 5. Conclusions

In this study, we identified 7581 intronless genes in the faba bean genome and found that a subset of these genes was responsive to salt stress. Expression analysis revealed clear cultivar- and tissue-dependent responses to NaCl treatment and highlighted *VfERF1A* and *VfHSP17.8* as candidate genes potentially involved in salt-stress responses. Predicted interaction networks, together with the LUC assay, supported a possible VfERF1A-related signaling context involving VfEIN2, although further functional validation is still required.

We further developed a *VfERF1A*-based KASP marker from an A/C SNP at chr4-919,763,421. In a 97-accession panel, the marker showed clear genotype clustering and was associated with multiple quantitative salt-response traits, including QYmax (Fv/Fm), relative shoot fresh weight, and relative plant height under salt treatment. Based on QYmax, the marker showed a preliminary predictive accuracy of 86.36% in this panel. Together, these results provide genomic resources, candidate genes, and a marker with potential utility for salt-stress response evaluation in faba bean. However, these findings should be interpreted cautiously, and further validation in independent populations, under diverse environmental conditions, and with additional functional evidence will be required before broader application can be established.

## Data Availability

Transcriptomic raw sequencing data have been deposited in the NCBI Sequence Read Archive database under accession number PRJNA1391386 (https://www.ncbi.nlm.nih.gov/bioproject/PRJNA1391386) (accessed on 23 December 2025).

## Supplementary Materials

The following supporting information can be downloaded at: https://www.mdpi.com/article/10.3390/genes17040381/s1, Figure S1. PCA of RNA-seq samples. Figure S2. Numbers of DEGs under salt stress. Figure S3. Heatmap of gene expression patterns. Figure S4. Preliminary yeast two-hybrid assay for selected candidate interactions of VfERF1A. Table S1. Summary of RNA-seq quality and mapping statistics for all libraries. Table S2. Primers used for qRT-PCR analysis. Table S3. Retained Arabidopsis protein IDs used for exploratory PPI analysis and their basis for inclusion. Table S4. Primers used for LCI vector construction. Table S5. KASP primers used for SNP genotyping. Table S6. Distribution of intronless genes in the Vicia faba genome. Table S7. FPKM expression matrix of all genes across samples in Vicia faba. Table S8. Summary of differentially expressed genes identified in the four pairwise comparisons. Table S9. Transcriptome expression levels of 15 common intronless DEGs. Table S10. Protein–protein interaction scores from STRING. Table S11. SNP information of *VfERF1A*. Table S12. KASP genotypes and salt-response traits in the faba bean panel.
