# Supplementary material for: Identification of Intronless Genes and the Development of KASP Markers for Salt Responses in Vicia faba L
Source: Genes (Basel). 2026 Mar 27;17(4):381. doi: 10.3390/genes17040381 (PMC13115572; doi:10.3390/genes17040381)
Supplement: Supplementary file 1 [file genes-17-00381-s001.zip › Supplementary Fig S1-S4.pdf]

Supplymentary Figure

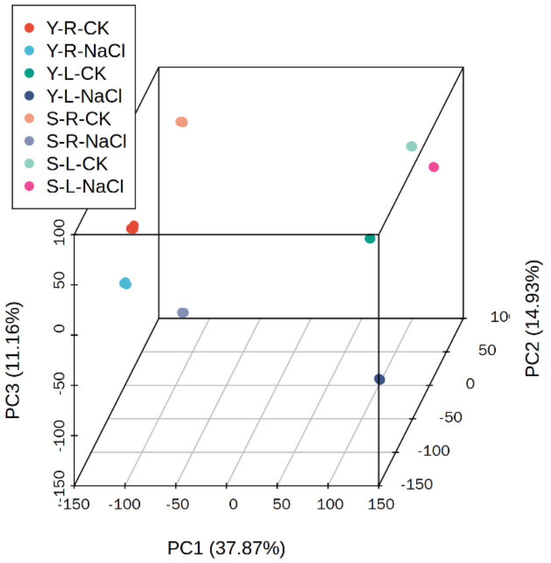

Fig S1. PCA of RNA-seq samples. Principal component analysis (PCA) of RNA-seq samples from Yundou 1183 (Y) and Sucan 4 (S) under control (CK) and salt treatment (NaCl) in roots (R) and leaves (L). The first three principal components (PC1, PC2, and PC3) explain 37.87%, 14.93%, and 11.16% of the total variance, respectively.

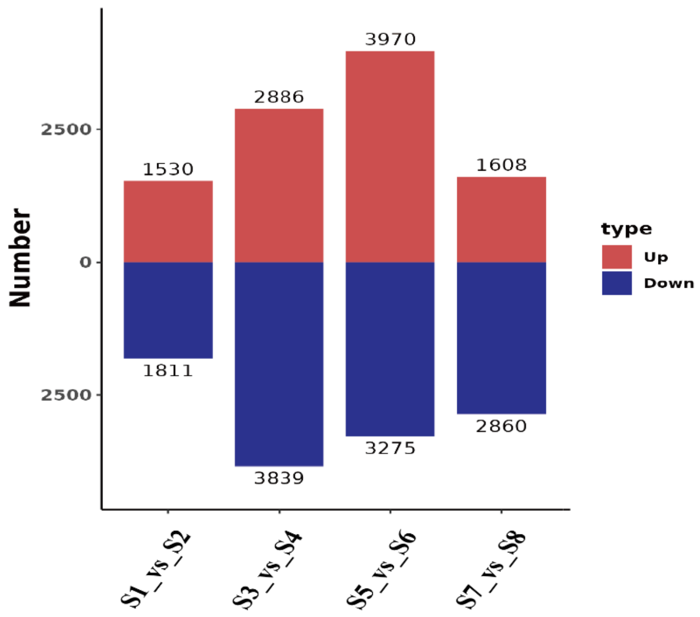

Fig S2. Numbers of DEGs under salt stress. Differentially expressed genes (DEGs) identified under salt treatment in roots and leaves of Yundou 1183 (YD1183) and Sucan 4 (Sucan4). Stacked bars show the numbers of upregulated (red) and downregulated (blue) DEGs for each comparison: S1 vs S2 (Y-R-CK vs Y-R-NaCl), S3 vs S4 (Y-L-CK vs Y-L-NaCl), S5 vs S6 (S-R-CK vs S-R-NaCl), and S7 vs S8 (S-L-CK vs S-L-NaCl).

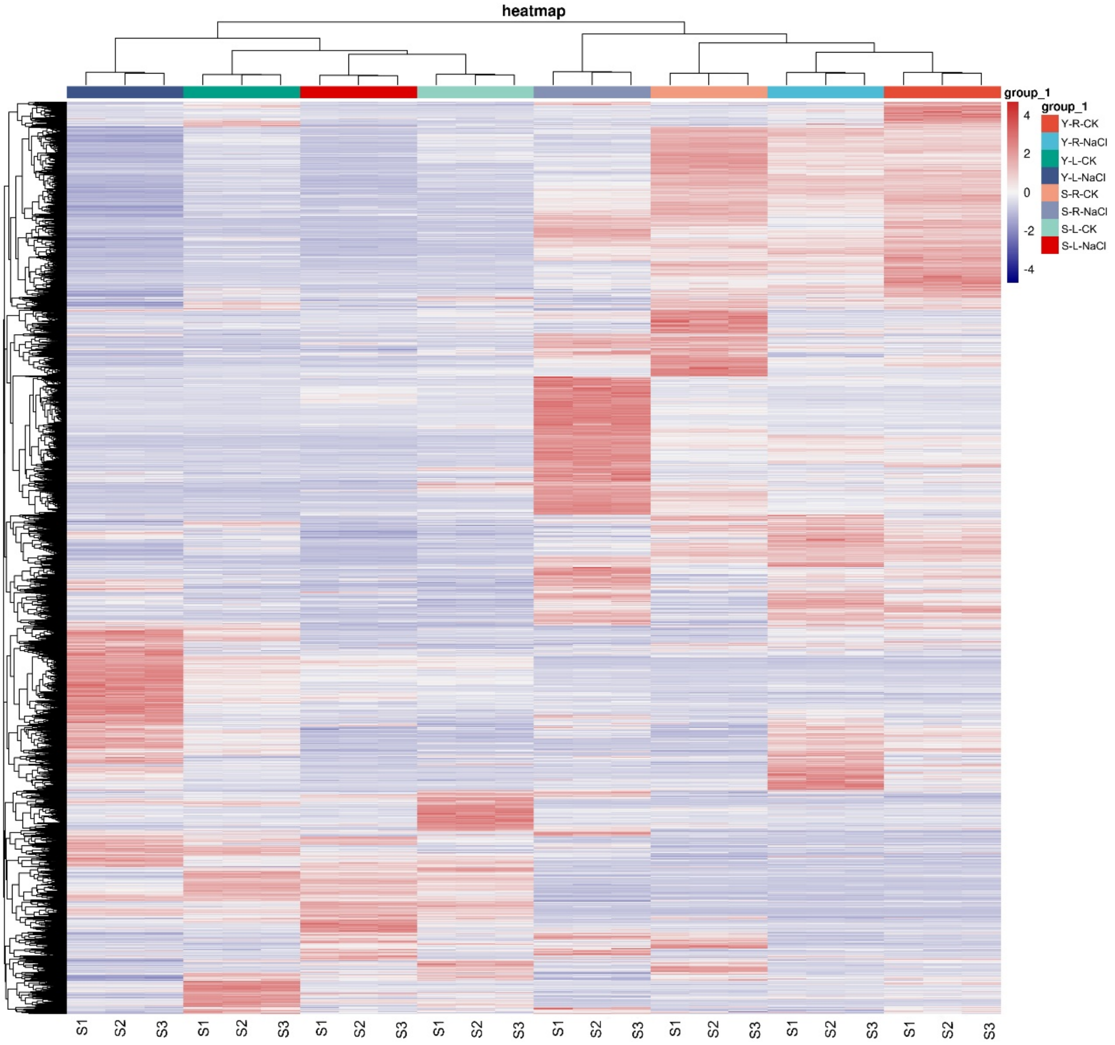

Fig S3. Heatmap of gene expression patterns. Red indicates higher expression and blue indicates lower expression. Sample groups are: Y-R-CK (S1), Y-R-NaCl (S2), Y-L-CK (S3), Y-L-NaCl (S4), S-R-CK (S5), S-R-NaCl (S6), S-L-CK (S7), and S-L-NaCl (S8).

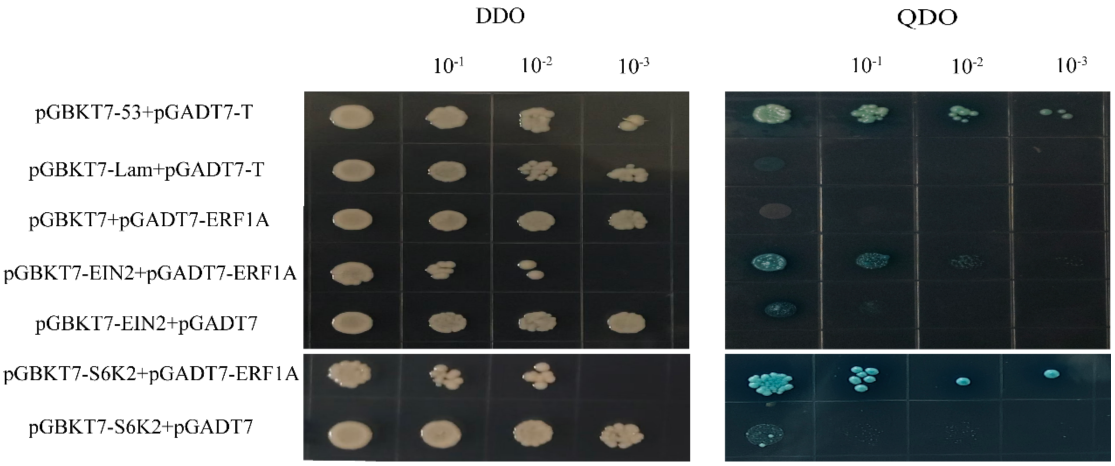

Fig. S4. Preliminary yeast two-hybrid assay for selected candidate interactions of VfERF1A.Yeast co-transformants were grown on SD/–Leu/–Trp (DDO) and SD/–Leu/–Trp/–His/–Ade (QDO) media at serial dilutions (10^-1, 10^-2, and 10^-3). The positive control (pGBKT7-53 + pGADT7-T) showed clear growth on QDO medium, while the negative control (pGBKT7-Lam + pGADT7-T) did not. The candidate combinations pGBKT7-EIN2 + pGADT7-ERF1A and pGBKT7-S6K2 + pGADT7-ERF1A also showed growth on QDO medium. However, weak background activation was detected in some control combinations, indicating possible autoactivation or nonspecific background. Accordingly, these data are provided only as supplementary and preliminary evidence and should be interpreted cautiously.
